# From dermatopathology to the courtroom—The promise of cutaneous molecular biomarkers

**DOI:** 10.3389/fmed.2026.1710443

**Published:** 2026-01-27

**Authors:** Fortunato Pititto, Elisa Paladini, Giuseppe Pulin, Roberto Bellacicco, Maricla Marrone

**Affiliations:** Forensic Medicine, Interdisciplinary Department of Medicine (DIM), Department of Forensic Medicine, University of Bari “Aldo Moro”, Bari, Italy

**Keywords:** ethical implications in forensic medicine, forensic dermatopathology, legal admissibility of evidence, microRNA, molecular biomarkers, post-mortem interval estimation, wound age determination, wound vitality assessment

## Introduction

The skin represents an organ of fundamental importance in forensic medicine, as it is frequently the site of injuries and signs of forensic relevance. Such lesions can be assessed not only macroscopically and histologically, but also through molecular biology techniques. While in the clinical setting the integration of morphology and molecular biology has revolutionized the diagnosis and prognosis of neoplastic and inflammatory diseases (1, 2), forensic applications remain at a very early stage.

The current convergence between these two fields raises a critical question: can molecular biomarkers, already established in dermatopathological practice, become reliable and legally admissible tools for wound dating and post-mortem interval estimation?

## From traditional markers to molecular dermatopathology

For many years, histological and immunohistochemical evaluation has been regarded as the standard for distinguishing vital from post-mortem lesions. Markers such as tryptase, IL-15, CD15, CD45, MMP-2, and MMP-9 have provided valuable insights, yet none has proven conclusive in cases of advanced decomposition (3, 4).

Heat shock proteins (HSPs), whose expression increases in response to traumatic or hypoxic stimuli, have further demonstrated their utility as indicators of lesion vitality and, in some instances, as potential tools for estimating wound age. Nevertheless, their use remains unsupported by definitive validation (5).

At the molecular level, an early contribution to the field was provided by Bauer et al. (6), who attempted to estimate the post-mortem interval (PMI) through quantification of mRNA degradation using multiplex Real Time- Polymerase Chain Reaction (RT-PCR) of the *fatty acid synthase* (FASN) gene. However, the wide confidence intervals observed, together with the unexpected stability of RNA within the first 48 h, limited the reliability of this approach, which remains confined to the experimental domain without practical forensic applicability.

Beyond RNA, DNA degradation has also been investigated as a potential marker of PMI, since it follows temporally consistent patterns. Yet, its applicability is significantly constrained by the scarcity of human samples with precisely documented PMI, by variability among tissues, and by the lack of validated multiparametric models (7, 8). In essence, these investigations have the merit of opening new perspectives, but they remain restricted to an exploratory stage.

## microRNA: promising yet still immature candidates

Among emerging biomarkers, microRNAs (miRNAs) have garnered increasing attention. These small non-coding RNAs are characterized by remarkable stability in formalin-fixed tissues and resistance to degradation, making them potentially suitable for post-mortem studies. They are expressed in both inflammatory and traumatic conditions and have already found established applications in cutaneous oncology.

Preliminary evidence suggests that miRNAs can discriminate between vital and post-mortem lesions. Neri and colleagues (9) identified significant differences in ligature marks from hanging cases, demonstrating that specific expression profiles are indicative of lesion vitality. Other studies have proposed that their stability may make them suitable for estimating the PMI, although the most promising markers to date have been identified not in the skin, but in tissues such as skeletal muscle, heart, and brain (10, 11). A recent systematic review further highlighted the potential of miRNAs for wound-age estimation, while also stressing the scarcity of original studies and the absence of multicenter validation (12).

Their biological stability, transferability from clinical experience, and potential for methodological standardization make miRNAs the most promising candidates for forensic dermatopathology. However, the degradation kinetics of specific miRNAs are influenced by multiple factors, including environmental conditions during sample preservation, the intrinsic properties of the tissue, and the amount of ribonucleases present in the matrix under study (10). Consequently, their premature introduction into judicial settings risks undermining their credibility: without international validation and methodological consensus, any result could be challenged or excluded in court proceedings.

## Ethical and legal considerations

The introduction of molecular biomarkers into forensic practice represents not only a scientific challenge but also an ethical and legal test.

A first issue concerns the concept of admissibility and evaluation of scientific evidence. According to internationally recognized legal standards, such as the Daubert criteria in the United States, a method must be validated, reproducible, and widely accepted within the scientific community in order to be deemed reliable. At present, no molecular approach applied to the skin—whether mRNA, miRNA, proteomic, or metabolomic—meets these requirements. Presenting such results in a courtroom would therefore likely lead to disputes over their reliability, with the consequent risk of undermining both the credibility of the expert and the discipline as a whole. This could significantly slow down, if not altogether hinder, the development of the field.

It is thus essential to establish shared protocols for the collection, preservation, and analysis of skin samples. In their absence, the results produced by different laboratories remain non-comparable, preventing the definition of universally accepted threshold values and limiting the transferability of data to forensic practice. Strengthening and creating international multicenter consortia and working groups dedicated to defining shared methodological criteria and quality standards is therefore indispensable. In legal terms, this relates directly to the concept of the *chain of custody*, which must be clearly defined and strictly regulated.

Another limitation lies in the scarcity of human samples with precisely documented times of death, as well as the significant ethical implications associated with their use. As a consequence, many findings still rely on animal models, whose translation into the human forensic setting remains an open issue.

Finally, an often-overlooked aspect is the technological availability of advanced molecular methods. Their implementation requires well-equipped laboratories, high costs, and specialized personnel. This raises the risk of creating a “two-tier justice system,” where advanced analyses are only accessible in centers with adequate resources, and where only litigants with sufficient financial means can rely on these techniques—ultimately weakening the principle of equality of arms in criminal proceedings. Standardization must therefore extend beyond methodology to encompass equity of access, ensuring that justice is not determined by technological or economic disparities.

## Discussion and conclusions

Molecular dermatopathology offers fascinating prospects for forensic medicine, but its greatest risk lies in premature adoption. Clinical experience demonstrates that consolidated biomarkers such as BRAF, NRAS, or PD-L1 required years of multicenter studies, harmonized protocols, and international guidelines before entering routine practice and being considered reliable for therapeutic decision-making. In forensic medicine, this path has only just begun, and it is evident that none of the currently proposed cutaneous biomarkers—whether mRNA, miRNA, proteomic, or metabolomic—meets the criteria for admissibility of scientific evidence according to international legal standards, particularly those based on the principle of “widespread acceptance.”

The introduction of methods lacking robust validation into the courtroom would risk opening the door to challenges, undermining the credibility of the expert, and, more broadly, eroding the trust of the judicial system in the scientific community. Among the available approaches, miRNAs emerge as the most promising candidates: their biological stability, together with the solid experience already gained in oncologic dermatopathology, places them in a privileged position. Yet, the absence of multicenter studies, rigorous standardization criteria, and interdisciplinary guidelines renders their forensic application still immature. The risk is two-fold: on one hand, systematic exclusion of such evidence from judicial proceedings, and on the other, their premature and superficial use leading to long-term delegitimization.

For these reasons, cutaneous molecular biomarkers should not yet be introduced into forensic routine or presented as courtroom evidence until a process of scientific consolidation is complete. This pathway must include the creation of international consortia dedicated to biomarker validation, the development of uniform protocols for sample collection and analysis, and, above all, the establishment of sustained dialogue among dermatopathologists, forensic pathologists, molecular biologists, and legal experts. Only through such efforts can innovation in this field secure a legitimate role in judicial settings without becoming a scientific and ethical boomerang.

In the coming years, research priorities should focus on key objectives. International multicenter validation of biomarkers must be the first step, through the coordinated collection of cutaneous samples from diverse traumatic and post-mortem contexts to reduce inter-laboratory variability. In parallel, methodological standardization will be required, with the definition of univocal procedures for sample retrieval, preservation, extraction, and biomarker analysis. Another avenue of development lies in the creation of shared digital repositories, encompassing both histopathological images and molecular profiles, accessible to the wider scientific community to enhance reproducibility and comparability of data.

Integration with clinical dermatopathology will also be crucial: validation models already tested in cutaneous oncology may provide valuable methodological frameworks for forensic applications. At the same time, the drafting of interdisciplinary guidelines that address not only scientific but also legal and ethical aspects will be indispensable to ensure future applicability. Artificial intelligence should be developed with transparency and explainability at its core, minimizing bias while maintaining interpretability in judicial contexts. Finally, investment in interdisciplinary training programs for dermatopathologists, forensic pathologists, and judges will be vital to build a common ground of expertise and a shared language between science and law.

In this perspective, the future of molecular dermatopathology in forensic practice depends not solely on technological progress, but on the ability of the scientific and legal communities to collaborate in developing tools that are at once scientifically sound, reproducible, and legitimately admissible in court.
